# Whole genome sequencing data of *Streptococcus pneumoniae* isolated from Indonesian population

**DOI:** 10.1016/j.dib.2024.110251

**Published:** 2024-02-27

**Authors:** Miftahuddin Majid Khoeri, Yustinus Maladan, Korrie Salsabila, Lindawati Alimsardjono, Naritha Vermasari, Iva Puspitasari, Rina Yunita, Wisnu Tafroji, Rosantia Sarassari, Ratna Fathma Sari, Sarah Azhari Balqis, Ghina Athyah Wahid, Diana Shinta Purwanto, Kuntjoro Harimurti, Amin Soebandrio, Dodi Safari

**Affiliations:** a Eijkman Research Center for Molecular Biology, National Research and Innovation Agency, Cibinong, West Java, Indonesia; b Doctoral program in Biomedical, Faculty of Medicine, University of Indonesia, Jakarta, Indonesia; c Soetomo Hospital, Surabaya, East Java, Indonesia; d Kariadi Hospital, Semarang, Central Java, Indonesia; e Haji Adam Malik Hospital, Medan, North Sumatera, Indonesia; f Department of Microbiology, Faculty of Medicine, Universitas Sumatera Utara, Medan, Indonesia; g Department of Biochemistry, Faculty of Medicine, Sam Ratulangi University, Manado, North Sulawesi, Indonesia; h Departement of Internal Medicine, Faculty of Medicine, University of Indonesia, Jakarta, Indonesia; i Departement of Clinical Microbiology, Faculty of Medicine, University of Indonesia, Jakarta, Indonesia

**Keywords:** *Streptococcus pneumoniae*, Whole-genome sequence, Pneumococcal conjugate vaccine, Hospitalized patient, Healthy children, Adult, Indonesia

## Abstract

*Streptococcus pneumoniae* is the leading cause of bacterial pneumonia, bacteremia, and meningitis. Indonesia introduced the pneumococcal conjugate vaccine (PCV) nationwide in 2022. In this study, we present whole genome sequence (WGS) data of 94 *S. pneumoniae* isolates that were obtained from hospitalized patients, healthy children, and adult groups from different regions prior to PCV program in Indonesia. DNA sequences of *S. pneumoniae* were obtained using the TruSeq Nano DNA kit (Illumina NovaSeq6000 Platform). The genome data of *S. pneumoniae* features a 1,969,562 bp to 2,741,371 bp circular chromosome with 39–40% G+C

content. The genome includes 1935–3319 coding sequences (CDS), 2 to 5 rRNA genes, 43 to 49 tRNA genes, and 56 to 71 ncRNA. These data will be useful for analyzing the serotype, sequence type, virulence genes, antimicrobial resistance genes, and the impact of pneumococcal vaccination in Indonesia. The FASTQ raw files of these sequences are available under BioProject accession number PRJNA995903 and Sequence Read Archive accession numbers SRR25316461-SRR25316554.

Specifications Table

**Table:** 

Subject	Bacterial Sequencing
Specific subject area	Genomics
Type of data	Complete genome sequence data in FASTA format, figure, and image
How data were acquired	Genome sequencing platform: Illumina NovaSeq6000 Genome annotation: ASA3P, an automatic and highly scalable assembly, annotation, and higher-level analysis pipeline (https://github.com/oschwengers/asap).
Data format	Raw sequences (FASTQ)
Parameters for data collection	Genomic DNA was extracted from purified cultures of S. pneumoniae.
Description of data collection	Whole-genome sequencing, assembly, and annotation
Data source location	Eijkman Research Center for Molecular Biology, National Research and Innovation Agency, Cibinong, West Java, Indonesia
Data accessibility	Raw data (FASTQ) files of S. pneumoniae have been deposited in the National Center for Biotechnology Information, https://www.ncbi.nlm.nih.gov/, under BioProject database: PRJNA995903 Sequence Read Archive (SRA) database: https://www.ncbi.nlm.nih.gov/sra/?term=PRJNA995903

## 1 Value of the Data

• Data can support a comparative genome study of *S. pneumoniae* isolated from patients and healthy people in different region of Indonesia.
• Data provides insight into the mechanism of antibiotic resistance and predicts the antimicrobial resistance profile for further drug development and disease treatment.
• Data provides a baseline data for pneumococcal vaccination impact in Indonesia.

## 2 Background

*Streptococcus pneumoniae* (*S. pneumoniae*) is a Gram-positive, lancet-shaped, diplococcus bacteria that is classified as a fastidious bacterium that can grow in a facultatively anaerob environment. This bacterium typically resides in the nasopharynx as normal flora and capable to breaching sterile body sites, giving rise to various infections including meningitis, bacteremia, and pneumonia [1]. The percentage of deaths caused by pneumonia in children under the age of five in Indonesia is 4% in 2021 [2].

A comprehensive whole-genome sequencing data of *S. pneumoniae* has never been conducted in Indonesia before, marking a critical gap in our understanding of its genetic diversity and its implications for public health. The acquisition of whole-genome sequencing data for *S. pneumoniae* in Indonesia holds immense promise. Unlocking the entire genetic code of the bacterium may unravel its evolutionary history, pinpoint virulence factors, and identify antibiotic-resistance genes, all of which are crucial for tailoring effective strategies to combat infections. This data-driven approach can enhance disease surveillance, facilitate the tracking of transmission routes, and aid in the selection of appropriate treatment regimens, ultimately contributing to more precise and timely interventions against *S. pneumoniae* infections in Indonesia [3]. Furthermore, monitoring and evaluation of data during vaccination and post-vaccination era is essential for assessing the effectiveness of the vaccine, detecting changes in disease trends, improving vaccine coverage and future vaccine development [4].

## 3 Data Description

**Fig. 1:**
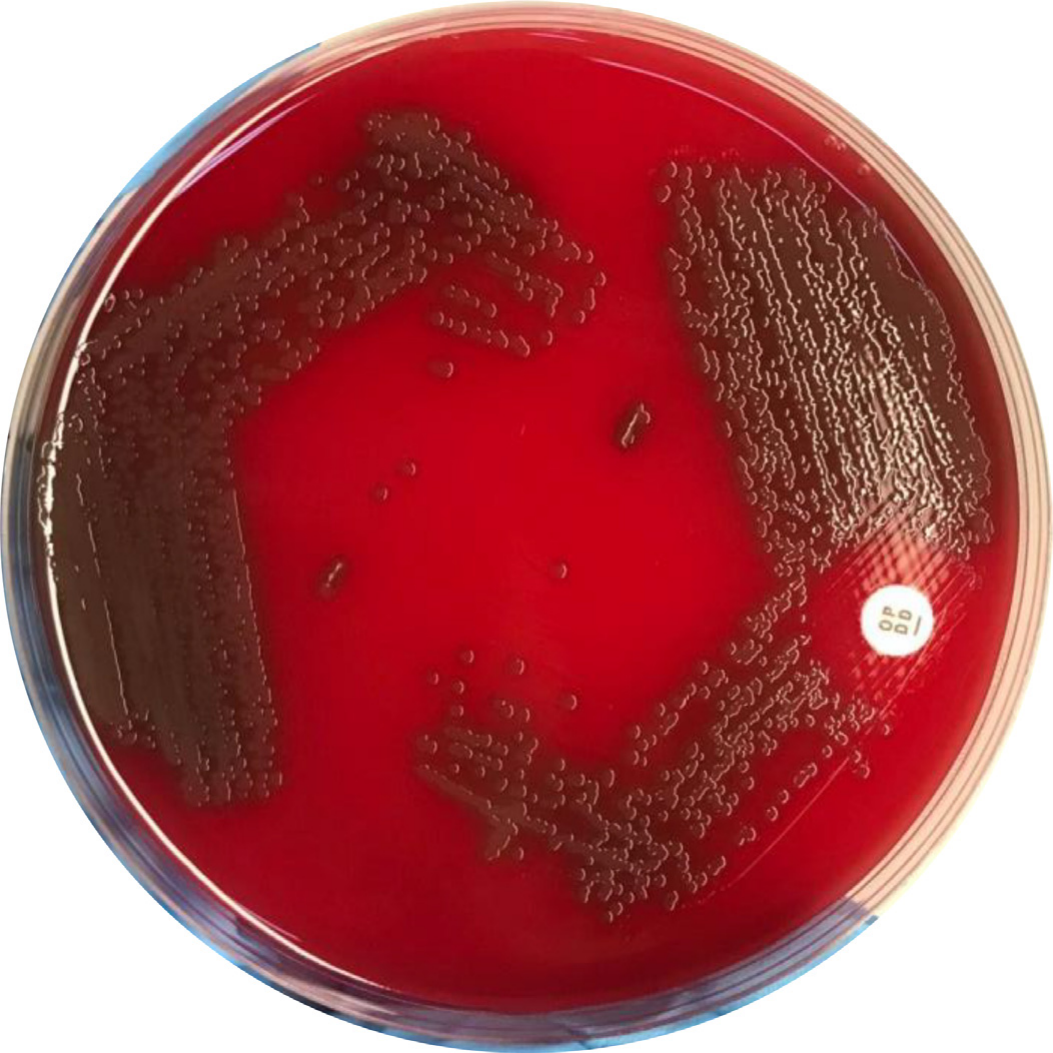
*Streptococcus pneumoniae* isolates from nasopharyngeal swab specimen. The isolates were susceptible to optochin susceptibility test (right side).

A total of 94 isolates of *S. pneumoniae* culture results (Fig. 1) were extracted and proceeded to the next-generation sequencing (NGS) process. These isolates were obtained from blood (*n* = 14), pleural fluid (*n* = 6), cerebrospinal fluid (*n* = 9), bronchoalveolar lavage fluid (*n* = 1), sputum (*n* = 7) and nasopharyngeal swab specimens (*n* = 45). The isolates were obtained from Banjarmasin (*n* = 4), Kotabaru (*n* = 9), Medan (*n* = 6), Surabaya (*n* = 34), Semarang (*n* = 10), Jakarta (*n* = 10), Lombok (*n* = 3), Makasar (*n* = 13), and Manado (*n* = 5), Indonesia as reported previously [5], [6], [7], [8]. All isolates were collected from 2012 to 2019.

**Fig. 2:**
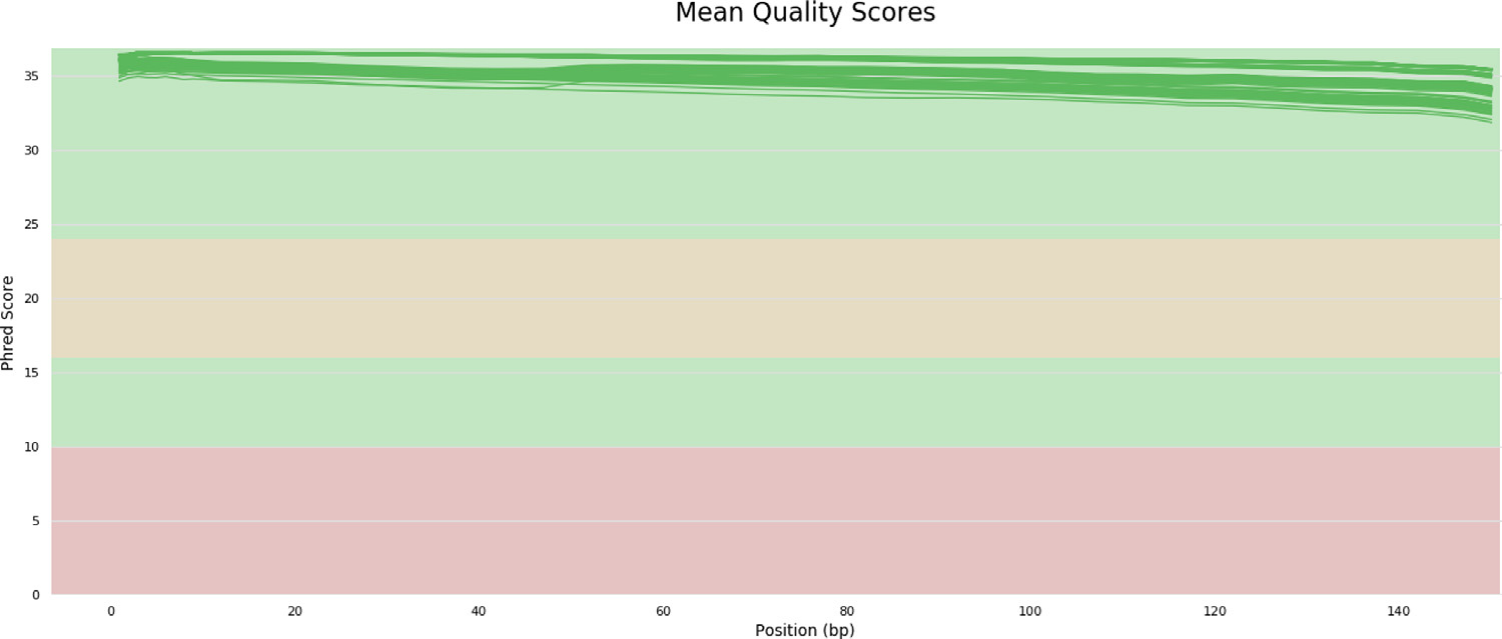
Whole genome sequencing quality control result. The Qscore measures the quality of the base calls in sequencing read, with higher Qscore indicates higher quality base calls. Qscore plot generated by FastQC shows the distribution of Qscore across all reads, *x*-axis representing the position in the read and the y-axis representing the Qscore. Green zone on Qscore plot indicates good quality score, meanwhile yellow indicating moderate quality scores, and red indicating poor quality scores.

**Fig. 3:**
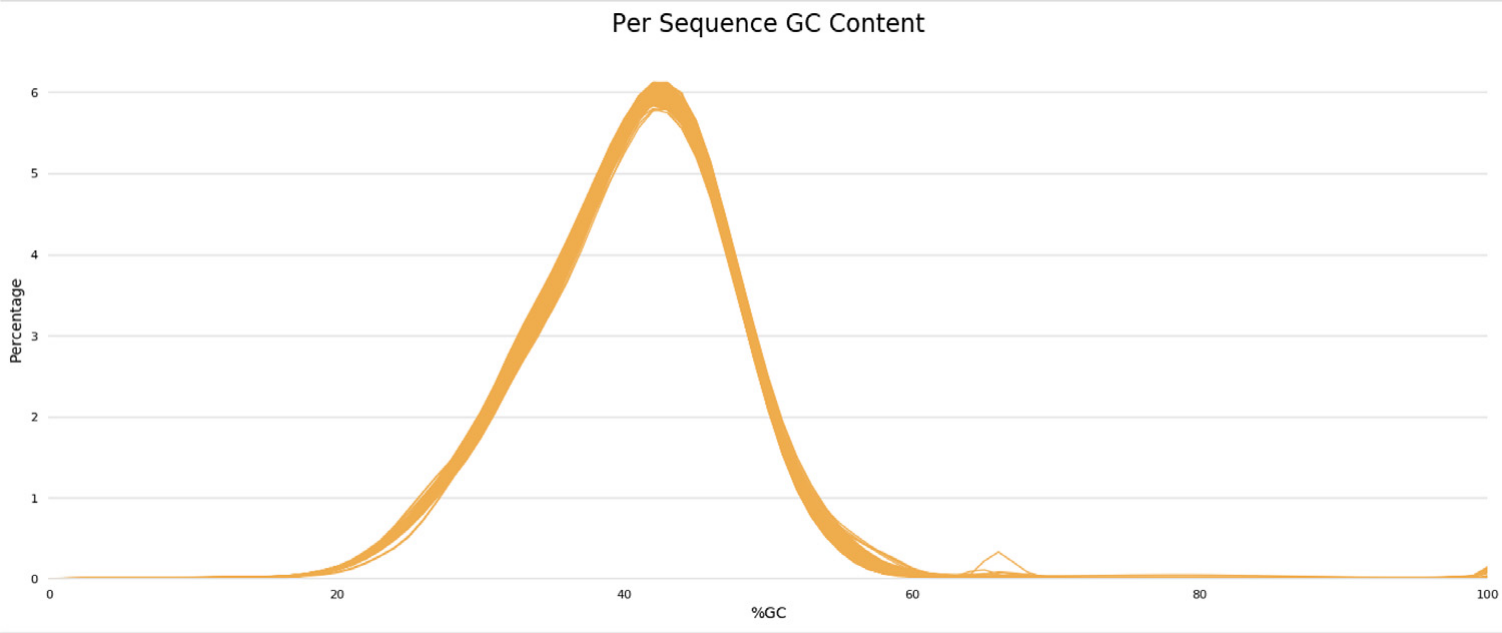
Percentage of GC content in each sample. The plot shows the distribution of GC content, *x*-axis representing the GC content and *y*-axis representing the number of sequences. The central peak in the plot corresponds to the overall GC content of the underlying genome. GC content of the central peak corresponds to the expected %GC for the organism, and the distribution should be normal unless there are over-represented sequences (sharp peaks on a normal distribution) or contamination with another organism (broad peak).

**Fig. 4:**
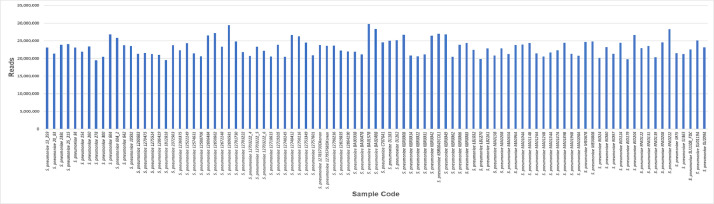
Total reads from 94 isolates of *Streptococcus pneumoniae*.

**Fig. 5:**
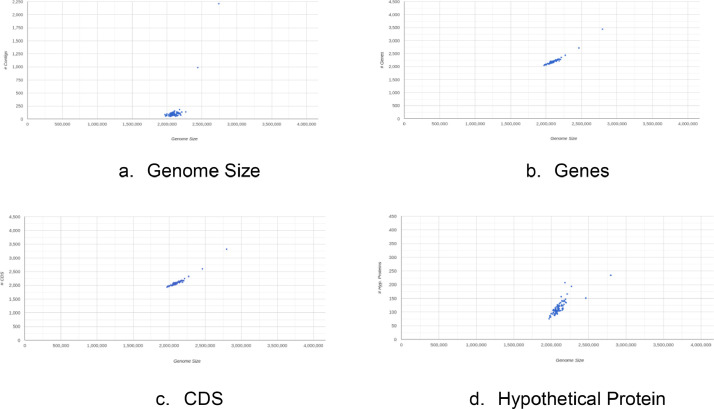
Genomic Profile after *de novo* assembly. The genome size of *S. pneumoniae* obtained ranged from 1,969,562 bp to 2,741,371 bp (a). The genome includes 2046–3441 genes (b) and 1935–3319 coding sequences (CDS; c) and the hypothetical protein range is 75–234 proteins (d).

Quality control results show that entire sample has a good quality with a mean of Qscore >30 (Fig. 2). GC content results from 94 samples are quite similar, ranging from 39 % to 40% (Fig. 3). Whole genome sequencing results generate millions of reads. The number of reads generated is 19,515,568-29,732,816 (Fig. 4). The assembly results showed that most of the samples had a genome size of around 2,000,000 bp (2 Mb). Two samples have size >2Mb (2.4 Mb and 2.75 Mb) (Fig. 5a). Most of the WGS results in this study showed a similar range of gene numbers (2046 to 3441 genes) (Fig. 5b). Furthermore, the number of Coding Sequences (CDS) is around 1935–3319 (Fig. 5c). The number of hypothetical proteins produced is 75 to 234 proteins (Fig. 5d). Based on the results of the Taxonomic Classification using K-mer/ANI and 16S rRNA, all samples were *S. pneumoniae* with the TaxID code 1313.

## 4 Experimental Design, Materials, and Methods

### 4.1 Bacterial culture

All *S. pneumoniae* isolates were stored in skim-milk-tryptone glucose glycerol (STGG) medium and stored at -80 °C. *S. pneumoniae* isolates were streaked into 5% sheep blood agar and incubated in 5% CO_2_ at 37 °C overnight. The isolate was examined for the appearance of alpha-hemolytic colonies and were identified by susceptibility to optochin (Fig. 1).

### 4.2 DNA extraction and library preparation

Genomic DNA was extracted from *S. pneumoniae* isolates using the DNeasy Blood & Tissue Kit (Qiagen, 69606) pre-treatment with mutanolysin and lysozyme. The *S. pneumoniae* genome was sequenced at PT Indolab Utama, Jakarta, Indonesia (Macrogen Co., Ltd., Singapore) using the Illumina platform. Library preparation was performed using the TruSeq Nano DNA Kit (Illumina, NE, USA) according to the manufacturer's instructions.

### 4.3 Sequencing analysis

The sequencing analysis is systematically evaluated using ASA3P pipeline (https://github.com/oschwengers/asap) [9]. ASA_3_P is a bionformatic tool designed for the pre-processing, assembly, annotation, and analysis of bacterial genomes data that incorporates several open source bioinformatic tools for transforming raw data sequencing into valuable information and insights. The quality of WGS data was assessed using FastQC (https://www.bioinformatics.babraham.ac.uk/projects/fastqc/">) and visualized with multiQC (https://multiqc.info/). Genome data assembly was performed by de novo assembly using the Automatic Bacterial Isolate Assembly, Annotation and Analyzes Pipeline (ASA_3_P) pipeline (https://github.com/oschwengers/asap) [9]. Reads that passed the quality control step were assembled into contigs using SPAdes. Afterwards, contigs will be arranged with a scaffolding process with ASA_3_P using Medusa tool (http://150.217.159.17/medusa/). Subsequently, ASA_3_P uses Prokka (https://github.com/tseemann/prokka) and Barrnap (https://github.com/tseemann/barrnap) to annotate contigs and scaffolds. ASA_3_P pipeline conduct taxonomy classification using Kmer/ANI method. Therefore, typing method for closely related bacterial strains within a species in ASA_3_P pipeline were performed by pubMLST (https://pubmlst.org/organisms/streptococcus-pneumoniae).

## Data Availability

Streptococcus pneumoniae Raw sequence reads (Original data) (NCBI).
